# Health literacy, illness perception, and their association with medication adherence in end-stage renal disease

**DOI:** 10.1007/s11255-025-04472-8

**Published:** 2025-04-04

**Authors:** Muhammad Amir Hamza, Shahan Ullah, Hina Ahsan, Wajeeha Ali, Mariam Masud, Ali Ahmed

**Affiliations:** 1 Department of Pharmacy, Faculty of Pharmaceutical Sciences, Riphah International University, Islamabad, Pakistan; 2 Department of Pharmacy Practice, Faculty of Pharmaceutical Sciences, Lahore University of Biological and Applied Sciences (UBAS), Lahore, Pakistan; 3 Department of Nephrology, Pakistan Institute of Medical Science (PIMS), Islamabad, Pakistan; 4 Division of Infectious Diseases and Global Public Health, School of Medicine, University of California San Diego (UCSD), La Jolla, CA USA

**Keywords:** Chronic kidney disease, End-stage renal disease, Adherence, Compliance, Medication

## Abstract

**Background:**

Medication non-adherence is a prevalent and complicated problem among patients receiving hemodialysis. Strictly following the prescribed medication regimen is crucial for achieving successful dialysis in end-stage renal disease.

**Objective:**

This study aimed to investigate how health literacy and illness perception influence medication adherence in hemodialysis patients.

**Methods:**

An observational cross-sectional study was conducted from October 2023 to February 2024 at dialysis units of multisite hospitals across Rawalpindi and Islamabad, Pakistan. A pre-validated, reliable, and interview-based questionnaire was utilized, and a convenience sampling technique was employed to collect data from 390 patients. The Statistical Package for the Social Sciences version 23.0 was utilized for statistical analysis.

**Results:**

More than half of the dialysis patients were female, 52.8%, 31% were between the ages of 46–60, and 66.6% had minimal or no education. Of the patients studied, 45.1% exhibited inadequate health literacy, 46.7% held negative perceptions of their illness, and 41.8% demonstrated low levels of medication adherence. The study found a weak correlation: a negative correlation between health literacy and illness perception (*r* = – 0.080), a positive correlation between health literacy and medication adherence (*r* = 0.024), and a negative correlation between illness perception and medication adherence (*r* = –0.061), none of which were statistically significant.

**Conclusion:**

A considerable proportion of the patients demonstrate low medication adherence, inadequate health literacy, and negative perceptions of their illness, highlighting the urgent need for targeted interventions. Structured educational programs, motivational interviewing, individual or group support, and in-person or remote (web- or telephone-based) counseling can help address these issues. Additionally, healthcare professionals-led interventions by doctors, pharmacists, and nurses play a crucial role in raising awareness, enhancing medication adherence, and optimizing treatment outcomes.

**Supplementary Information:**

The online version contains supplementary material available at 10.1007/s11255-025-04472-8.

## Introduction

Chronic kidney disease (CKD) has a global prevalence of 9.1%, affecting approximately 700 million people [1, 2]. It affects 434.3 million adults in Asia; 5.6 million have end-stage CKD [3]. In Pakistan, 21.2% of the population suffers from CKD, with a higher prevalence (43.6%) among individuals above 50 years of age [4]. CKD is a chronic condition affecting kidneys where the glomerular filtration rate (GFR) decreases to less than 60 ml/min/1.73 m^2^ for at least three or more months [5, 6]. CKD can progress to end-stage renal disease (ESRD) if left untreated and requires renal replacement therapy to supplement or replace the null functioning of kidneys [7]. Hemodialysis is a life-sustaining treatment for ESRD patients; however, several complications may occur as an outcome, such as an increased risk of infections and cardiovascular events. The success of treatment heavily relies on the management of adverse events through medication adherence [7].

The World Health Organization (WHO) defines treatment adherence as the extent to which the patient’s actions are acceptable, based on the guidance provided by healthcare providers [8]. Adherence to medications ensures the effective removal of toxins, effective management of comorbid conditions, and prevention of infection and cardiovascular events in CKD patients, whereas non-adherence leads to life-threatening complications such as fluid overload and hyperkalemia [9, 10]. Therefore, strict medication adherence is important to improve the prognosis in CKD patients.

The medication non-adherence rates among hemodialysis patients with CKD range from 17.2% to 69% globally [11–15]. In Pakistan, poor adherence is reported in 52–59% of the CKD patients [16]. Several patient-level and system-level factors affect medication adherence among chronic patients, where lack of knowledge regarding medication use and poor patient-provider communication majorly hinder medication adherence [17]. Additionally, CKD patients face a heavy pill burden with 14–15 medicines to manage their comorbidities, which results in non-adherence [17]. Moreover, several socioeconomic and demographic factors such as education level, employment status, income level, marital status, and medication costs and availability also affect medication adherence in patients [18–21].

Health literacy is a cognitive factor related to individuals’ ability to utilize health information and make informed decisions [22]. Health literacy has been shown to improve medication adherence significantly. For instance, in a study conducted in Korea, health literacy was found to be a positive predictor of medication adherence in chronic disease patients [23]. Furthermore, illness perception, a related concept that refers to a patient’s health-related behavior and concepts in chronic diseases, also significantly influences patients’ adherence to diet and medicines but needs research attention for its impact on treatment adherence in CKD patients [9].

Despite the significance of health literacy and illness perception in chronic disease management, limited research is available on the combined predictive ability of these measures on medication adherence in hemodialysis patients. In a study, health literacy and illness perception predicted self-management scores by 45% among hemodialysis patients in Indonesia [21]. Similarly, Marshall et al. found that health literacy and illness perception significantly increased medication adherence in renal dialysis patients [24]. However, these studies did not evaluate the combined effect of these measures on medication adherence and health outcomes. A comprehensive evaluation of predictive factors of medication adherence among CKD patients will be crucial in improving patients’ health outcomes by guiding targeted interventions and healthcare policies.

This study investigates the relationships between health literacy, illness perception, and medication adherence among hemodialysis patients in Pakistan. While previous studies have explored patients’ illness perceptions [25–28] and medication knowledge [16, 29], the role of health literacy in medication adherence remains unexplored. Given the socioeconomic challenges faced by many dialysis patients, including low income, limited education, and older age, this study also assesses how these characteristics impact adherence to prescribed medication.

### Methodology

The study was reported utilizing the Strengthening the Reporting of Observational Studies in Epidemiology (STROBE) checklist in compliance with the guidelines of the Equator network [30].

### Ethical consideration

The study was conducted in compliance with the guidelines outlined in the Declaration of Helsinki [31] and obtained approval from the Research Ethics Committee of Riphah Institute of Pharmaceutical Sciences, Riphah International University, Islamabad, Pakistan (Reference Number REC/RIPS/27). The participants were provided with detailed information regarding the objective and methods of the study through a comprehensive written consent form, which included explanations of the study’s purpose, procedures, potential risks, and benefits. Participants were allowed to ask questions, and their concerns were addressed to ensure full understanding. Verbal authorization was acquired from each participant after confirming their comprehension of the study details and emphasizing that their participation was voluntary and that they could withdraw at any time without any consequences.

### Study design and study setting

A cross-sectional study was conducted on hemodialysis patients from October 2023 to January 2024 at the nephrology departments of multiple institutions in Rawalpindi and Islamabad. These institutions include the Pakistan Institute of Medical Sciences (PIMS), Quaid Azam International Hospital (QIH), Riphah International Hospital (RIH) in Islamabad, as well as the Fauji Foundation Hospital and Railway Hospital in Rawalpindi.

Rawalpindi and Islamabad are termed Twin cities and are situated in the northeast part of Pakistan. They are among the most populated urban areas in the country, with a combined population of approximately 2.3 million [32]. These institutions serve patients from many ethnic and socioeconomic backgrounds. They were chosen based on their large number of patients and varied demographics, ensuring a sample accurately representing the entire population [33]. Moreover, being prominent healthcare institutions in the area, these hospitals provide extensive nephrology services, ensuring convenient access for a diverse population of hemodialysis patients [34].

### Study population

The study population was CKD patients undergoing hemodialysis treatment who were selected from various dialysis units based on the study’s inclusion and exclusion criteria. To be eligible, the participants had to be at least 18 years old, have a confirmed diagnosis of ESRD, have been receiving hemodialysis therapy for over 6 months, be in stable condition, and provide informed consent. Participants with visual, hearing, or cognitive problems and those unable to complete surveys were excluded. Additionally, the study did not include patients with psychiatric disorders, pregnant individuals, or those with severe comorbid conditions.

### Sample size and sampling technique

The online Rao soft calculator [35] was used to compute the sample size, employing a 5% margin of error, a 95% confidence interval, and a 50% response rate. This calculation indicated that a total sample size of 377 participants was required. To ensure an adequate sample size, 420 patients were approached and voluntarily interviewed, with 390 ultimately included in the final analysis. A non-probability sampling method, specifically the convenience sampling technique, was utilized due to the constraints of gathering data from a specific cohort of dialysis patients at certain times and locations.

### Data collection tool and procedure

A structured and standardized 16-item Health Literacy questionnaire (HLQ), the Brief Illness Perception Questionnaire (BIPQ), and the 8-item Morisky Medication Adherence Scale (MMAS- 8) were used in this study. These scales were originally developed in English: the HLQ by Chew et al., [36] the BIPQ by Broadbent et al., [37] and the MMAS- 8 by Morisky et al. [38]. These scales are generic instruments that have been translated and validated in English. They are not specific to any particular disease or treatment group. Even though the HLQ, BIPQ, and MMAS- 8 are designed for self-administration, we used these as interview-based questionnaires to ensure better data quality, as some patients in the research setting may not be able to read and write.

The questionnaires were translated into Urdu using the World Health Organization’s translation guidelines. Urdu versions of the questionnaires were used to ensure comprehension among participants. To maintain the integrity of the original content, we followed a rigorous translation process. A team of bilingual experts conducted a forward translation from English to Urdu, followed by an independent translator’s backward translation to English to verify accuracy and conceptual equivalence. Pharmacy practice specialists and nephrologists carefully reviewed the translated versions to assess clarity and contextual relevance. Additionally, a pilot study was conducted with 25 participants to evaluate the readability, clarity, and applicability of the questionnaire. Feedback from the pilot study was incorporated, and the final refined version was used for data collection.

The questionnaire consisted of four sections. Section “Introduction” measured the patient’s sociodemographic characteristics (age, gender, marital status, educational background, current employment status, family income, and ethnicity) and clinical characteristics (years on dialysis, frequency of dialysis, daily pill burden, and comorbidities). Section “Methodology” measured health literacy using the HLQ. The assessment consists of 16 items that assess patients’ proficiency in reading and writing content regarding their health. Section “Results” measured illness perception using the BIPQ. It comprises nine questions that assess the eight domains of illness perception, including consequences, timeline, personal control, treatment control, identity, concern, understanding, and emotional response.

Section “Discussion” measured medication adherence using the MMAS- 8. It comprises eight items. This metric was developed to facilitate the identification of barriers and facilitators associated with adherence to long-term medication regimens. It encompasses both intentional and unintentional behaviors related to the use of medications, including scenarios where medications are not taken due to adverse effects or are forgotten. The author visited the dialysis unit and approached patients who met the inclusion and exclusion criteria. After obtaining informed consent, the selected patients were included in the study. The author personally administered the questionnaires and recorded the responses. Each participant’s session to go through and respond to the questionnaire took approximately 15–20 min.

### Scoring of tools

#### Scoring of health literacy

Health Literacy questionnaire consists of 16 items. The tool evaluates each question on a Likert scale with five points, ranging from 0 (always) to 4 (never), excluding questions 1–4, 14, and 15. These questions were coded in reverse order to determine the degrees of health literacy. The scores varied from 0 to 64, and the original scale categorized health literacy into three levels: inadequate (0–34), marginal (35–42), and adequate (43–64). However, for data analysis, we dichotomized health literacy by combining the marginal and adequate literacy categories (35–64) into a single group labeled ‘adequate health literacy.’ This approach has been widely used in previous research [39–41] to simplify statistical interpretation and provide clearer insights into health outcomes.

#### Scoring of illness perception

Illness perception has nine questions, eight rated on a scale of 0–10, and one open-ended question concerning the perceived cause of illness. High scores on personal control, treatment control, and understanding indicate positive perceptions, whereas higher scores on all the other domains indicate negative perceptions. The overall perception of illness is determined by inverting the scores of personal controls, treatment control, and understanding. The sum of the first eight items is then calculated, with higher values showing a negative perception and a lower value showing a positive perception. The median score for each domain is 5. If the participant's score is 5 or lower, they are categorized as having a positive perception. If the value exceeds 5, it is seen as indicative of a negative perception. The maximum score for total illness perception is 80. The study’s median score is 49. Participants scoring 49 or below are categorized as having a positive overall perception, while those scoring above 49 have a negative overall perception. The cut-off for negative illness perception was determined based on the median score of study populations, consistent with previous research conducted on chronic disease populations [42, 43], where the median has been used as a natural division point.

#### Scoring of medication adherence

The MMAS- 8 consists of 8 items. Responses for items 1–7 are dichotomous (“yes” = 0 points, “no” = 1 point), except for item 5, where “yes” scores 1 point and “no” scores 0 points (reverse-scored). Item 8 uses a 5-point Likert scale: 1 = never/rarely, 0.75 = once in a while, 0.50 = sometimes, and 0.25 = usually, with 0 corresponding to all the time. The total score ranges from 0 to 8, where higher scores indicate higher levels of adherence: 8 signifies significant adherence, 6–7 indicates moderate adherence, and below 6 suggests poor adherence.

### Statistical analysis

The questionnaires were checked for completeness and data were entered and analyzed using Statistical Package for Social Sciences (SPSS) version 23. The demographic, clinical characteristics, and scoring of health literacy, illness perception, and medication adherence were described through descriptive statistics. The categorical variables were displayed in percentages and frequencies, while means and standard deviations were calculated for continuous variables. The Shapiro–Wilk test and Kolmogorov–Smirnov test were used to check the normality of data. Spearman’s rank correlation coefficient was used to examine the relationship between health literacy, illness perception, and medication adherence scores. Univariate analysis was performed using the chi-square test, and multivariate analysis was performed using binary and ordinal regression analysis. A 95% confidence interval and a *p* value of < 0.05 were considered statistically significant.

## Results

A total of 420 hemodialysis patients were initially approached for the study. Of these, 390 met the eligibility criteria and agreed to participate. Twenty patients declined to be interviewed and the remaining ten were excluded due to incomplete data or health instability during the interview period. Consequently, the response rate for the study was approximately 90.7%.

### Demographics and clinical characteristics of hemodialysis patients

More than half of the study population was comprised of females (52.8%), married (73.6%), and old ages between 40 and 60 years (39%). Most of the respondents had spent 2–3 years on dialysis (28.7%), with a dialysis frequency of twice a week (84.4%). Further, most of the study population were suffering from 2 to 3 comorbid diseases (34.4%) and took 6 to 10 tablets on average (54.4%). The demographic and clinical characteristics of the study population are given in Table 1.

**Table 1:** Demographic and clinical characteristics of patients

Categories	*n* (%)
Age (years)	
18–30	58 (14.9)
31–45	76 (19.5)
46–60	152 (39.0)
≥ 61	104 (26.7)
Gender	
Male	184 (47.2)
Female	206 (52.8)
Marital status	
Single	61 (15.6)
Married	287 (73.6)
Others (divorced, widowed and widower)	42 (10.7)
Qualification	
Uneducated	98 (25.1)
Secondary education	162 (41.5)
Tertiary education	130 (33.3)
Current employment status	
Un-employed	86 (22.1)
Employed	35 (9.0)
Others (retried, self-employed, and housewives)	269 (68.9)
Family income (Pakistani rupees)	
10,000–30,000	53 (13.6)
31,000–50,000	150 (38.5)
51,000–100,000	175 (44.9)
> 100,000	12 (3.1)
Years on dialysis	
≤ 1	177 (45.4)
2–3	112 (28.7)
≥ 4	101 (25.9)
Frequency of hemodialysis	
Once a week	22 (5.6)
Twice a week	329 (84.4)
Thrice a week	39 (10.0)
Daily pills burden	
1–5	179 (45.9)
6–10	141 (36.2)
> 10	70 (17.9)
Comorbidity	
None	24 (6.2)
One disease	140 (35.9)
2–3 diseases	212 (54.4)
Four diseases	14 (3.6)
Ethnicity	
Urdu speaking	62 (15.9)
Punjabi speaking	225 (57.7)
Pashtun speaking	34 (8.7)
Sindhi speaking	5 (1.3)
Hindko speaking	29 (7.4)
Kashmiri speaking	35 (9.0)
Health literacy	
Inadequate	176 (45.1)
Adequate	214 (54.9)
Illness perception	
Negative perception	182 (46.7)
Positive perception	208 (53.3)
Medication adherence	
Low	163 (41.8)
Medium	141 (36.2)
High	86 (22.1)

### Health literacy score of hemodialysis patients

The median health literacy score of hemodialysis was 36 (28–45), where 54.9% of patients had adequate health literacy at 43 (38–53) and 45.1% of the patients had an inadequate health literacy score at 28 (23.25–32). Most participants found healthcare materials easy to understand and reported patient educational materials and medication labels as easier to understand, whereas hospital signs and medication bottle labels were reported to be more challenging to understand. A detailed account of health literacy among respondents is given in Table 2.

**Table 2:** The health literacy level of dialysis patients

Health literacy questions	Always *n* (%)	Often *n* (%)	Sometimes *n* (%)	Occasionally *n* (%)	Never *n* (%)
How often are appointment slips written in a way that is easy to read and understand? [R]	111 (28.5)	99 (25.4)	80 (20.5)	60 (15.4)	40 (10.3)
How often are medical forms written in a way that is easy to read and understand? [R]	98 (25.1)	108 (27.7)	79 (20.3)	59 (15.1)	46 (11.8)
How often are medication labels written in a way that is easy to read and understand? [R]	101 (25.9)	99 (25.4)	88 (22.6)	60 (15.4)	42 (10.8)
How often are patient educational materials written in a way that is easy to read and understand? [R]	81 (20.8)	120 (30.8)	91(23.3)	63 (16.2)	35 (9.0)
How often are hospital or clinic signs challenging to understand?	31 (7.9)	82 (21.0)	122 (31.3)	99 (25.4)	56 (14.4)
How often are appointment slips challenging to understand?	42 (10.8)	83 (21.3)	87 (22.3)	93 (23.8)	85 (21.8)
How often are medical forms challenging to understand and fill out?	44 (11.3)	87 (22.3)	79 (20.3)	97 (24.9)	83 (21.3)
How often are directions on medication bottles challenging to understand?	41 (10.5)	89 (22.8)	108 (27.7)	97 (24.9)	55 (14.1)
How often do you have difficulty understanding written information your health care provider (like a doctor, nurse, or nurse practitioner) gives you?	23 (5.9)	51 (13.1)	102 (26.2)	116 (29.7)	98 (25.1)
How often do you have problems getting to your clinic appointments at the right time because of difficulty understanding written instructions?	9 (2.3)	44 (11.3)	70 (17.9)	51 (13.1)	216 (55.4)
How often do you have problems completing medical forms because of difficulty understanding the instructions?	39 (10.0)	73 (18.7)	101 (25.9)	98 (25.1)	79 (20.3)
How often do you have problems learning about your medical condition because of difficulty understanding written information?	30 (7.7)	75 (19.2)	131 (33.6)	87 (22.3)	67 (17.2)
How often are you unsure how to take your medication(s) correctly because of problems understanding written instructions on the bottle label?	17 (4.4)	68 (17.4)	109 (27.9)	71 (18.2)	125 (32.1)
How confident are you in filling out medical forms by yourself? [R]	66 (16.9)	89 (22.8)	103 (26.4)	85 (21.8)	47 (12.1)
How confident do you feel you can follow the instructions on the label of a medication bottle?	54 (13.8)	106 (27.2)	111 (28.5)	87 (22.3)	32 (8.2)
How often can someone (like a family member, friend, hospital/clinic worker, or caregiver) help you read hospital materials? [R]	113 (29.0)	104 (26.7)	94 (24.1)	52 (13.3)	27 (6.9)

[R] reverse scoring

### Illness perception of hemodialysis patients

**Fig. 1:**
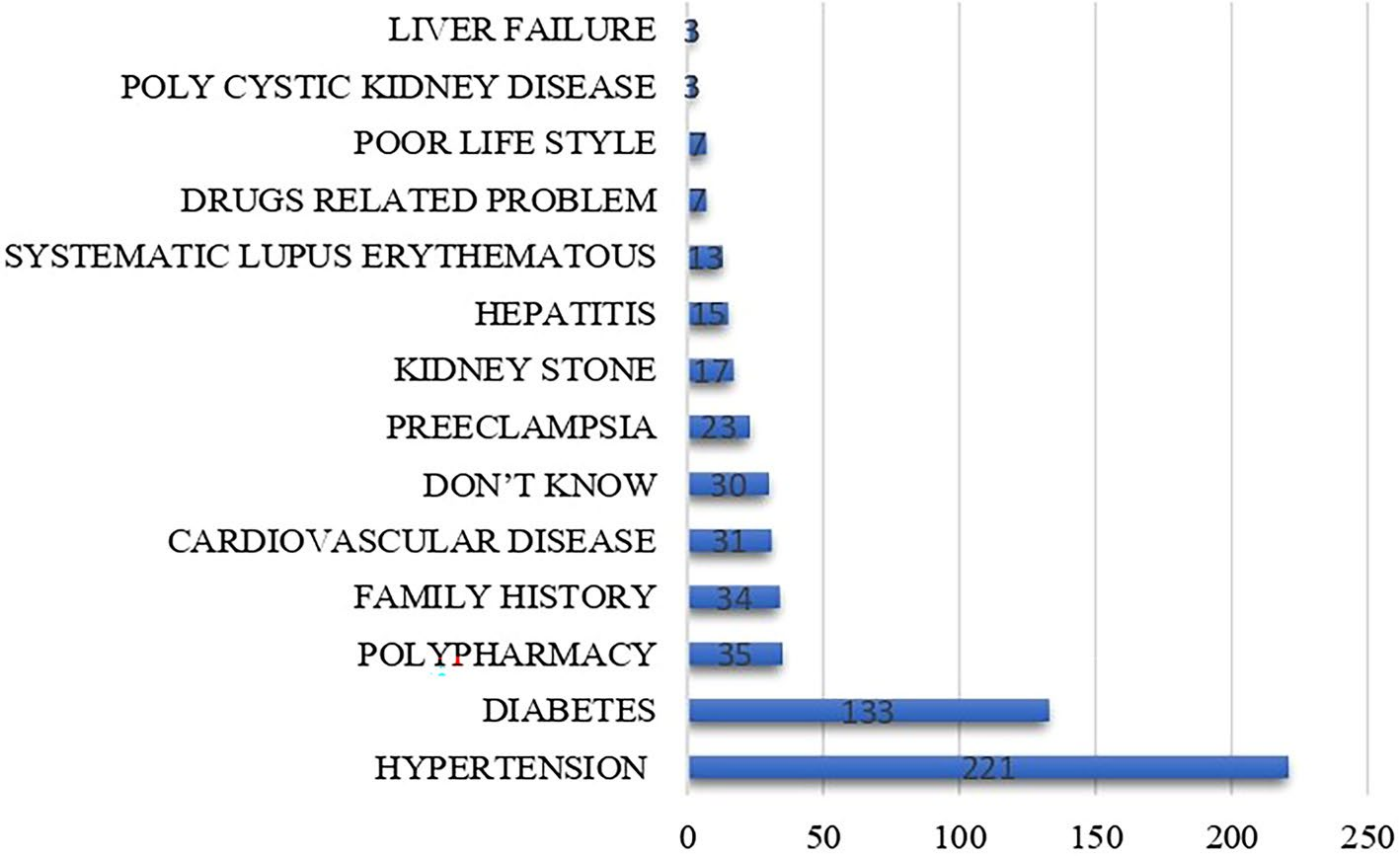
Patient perceptions of chronic kidney disease failure cauality factors

The mean and median illness perception scores were 49.3 ± 6.9 and 49 (45–54), respectively. Among the patients, 53.3% had positive illness perceptions with a mean score of 44.2 ± 4.1, while 46.7% had negative illness perceptions with a mean score of 55.1 ± 4.4. The most prevalent positive perception was regarding personal control, while the most prevalent negative perceptions were about concern and consequence followed by timeline. The mean perception scores and number of patients with positive and negative perceptions are given in Table 3. Item 9, an open-ended question, asked participants to identify the three most important factors they believed caused their illness. All 390 participants responded, providing a total of 569 answers. The most important perceived causes were hypertension (221 responses, 38.8%), followed by diabetes (133 responses, 23.3%), and polypharmacy (35 responses, 6.15%) (see Fig. 1).

**Table 3:** Illness perceptions of dialysis patients

Statements	Items	Mean ± SD	Median (IQR)	Positive perception *n* (%)	Negative perception *n* (%)
How much does your illness affect your life?	Consequence	8.96 ± 1.57	10 (8–10)	23 (5.9)	367 (94.1)
How long do you think your illness will continue?	Timeline	8.94 ± 1.77	10 (8–10)	29 (7.4)	361 (92.6)
How much control do you feel you have over your illness? [R]	Personal control	7.48 ± 2.06	8 (6–9)	317 (81.3)	73 (18.7)
How much do you think your treatment can help your illness? [R]	Treatment control	7.51 ± 1.27	8 (7–8)	348 (89.2)	42 (10.8)
How much do you experience symptoms from your illness?	Identity	6.58 ± 2.32	7 (5–9)	142 (36.4)	248 (63.6)
How concerned are you about your illness?	Concern	8.83 ± 1.52	9 (8–10)	21 (5.4)	369 (94.6)
How well do you feel you understand your illness? [R]	Coherence	6.71 ± 1.76	7 (5–8)	280 (71.8)	110 (28.2)
How much does your illness affect you emotionally? (e.g., makes you angry, scared, upset, or depressed?	Emotional representation	7.71 ± 2.41	8 (5.75–10)	97 (24.9)	293 (75.1)
Total		49.33 ± 6.95	49 (45–54)	208 (53.3)	182 (46.7)

[R] reverse scoring

### Medication adherence among hemodialysis patients

The overall median medication adherence score of hemodialysis patients was 6.75 (4.50–7.75), where a majority of patients 41.8% had low medication adherence scores with a median value of 3.75 (2.25–5) and 36.2%, and 22.1% of patients had medium 7 (6.75–7.25) and high adherence scores 8 (8–8), respectively. The responses to Morisky medication adherence scales and overall medication adherence scores are given in Table 4. Further, most of the patients found it challenging to remember to take all their medicine either all the time (46.7%) or usually (26.2%), followed by sometimes (18.7%), once in a while (5.4%), and often (3.1%).

**Table 4:** Medication adherence in dialysis patients

Questions of medication adherence	Yes	No
	*n* (%)	*n* (%)
Do you sometimes forget to take your medication?	147 (37.7)	243 (62.3)
Over the past 2 weeks, were there any days that you did not take your medication?	101 (25.9)	289 (74.1)
Have you ever cut back or stopped taking your medication without telling your doctor because you felt worse when you took it?	107 (27.4)	283 (72.6)
When you travel or leave home, do you sometimes forget to bring your medication?	66 (16.9)	324 (83.1)
Did you take your medication yesterday? [R]	347 (89.0)	43 (11.0)
When you feel that your health concern is under control, do you sometimes stop taking your medication?	95 (24.4)	295 (75.6)
Taking medication every day is a real inconvenience for some people. Do you ever feel hassled about sticking to your treatment plan?	209 (53.6)	181 (46.4)

Question no.8 of Medication adherence					
Question no.8	Never/Rarely *n* (%)	Once in a while *n* (%)	Sometime *n* (%)	Usually *n* (%)	All the time *n* (%)
How often do you have difficulty remembering to take all your medication?	12 (3.1)	21 (5.4)	73 (18.7)	102 (26.2)	182 (46.7)

[R] reverse scoring

### Correlations between health literacy, illness perception, and medication adherence

There was a weak negative correlation between health literacy and illness perception (*r* = − 0.080). This suggests that illness perception scores decrease slightly as health literacy scores increase. Health literacy showed a weak positive correlation with medication adherence (*r* = 0.024). This indicates that higher health literacy is slightly associated with better medication adherence. However, this correlation was not statistically significant (*r* = 0.631). Illness perception was weakly and negatively correlated with medication adherence (*r* = − 0.061). This implies that medication adherence tends to decrease slightly as illness perception scores increase. However, all of these correlations were not statistically significant (*p* > 0.05) (Table 5).

**Table 5:** Spearman correlation between health literacy, illness perception, and medication adherence

Variables	Health literacy	Illness perception	Medication adherence
Health literacy	–		
Illness perception	–0.080 (0.115)	–	
Medication adherence	0.024 (0.631)	–0.061 (0.229)	–

### Impact of demographic and clinical parameters on health literacy of the patients

The median adequate health literacy scores of patients who had acquired higher education (college/bachelor/postgraduate) were significantly higher than those who had acquired secondary/high school education or uneducated with median (IQR) values [44.5 (36–54) vs 33.0 (27–40) and 32.0 (24–40), *p* < 0.0001]. Similarly, the median (IQR) adequate health literacy scores were higher for a more significant number of years on dialysis, where patients receiving dialysis for 2–3 years had higher health literacy than those receiving for 1 year with median (IQR) scores [37.5 (31–49.7) vs 33.0 (26–40.5), *p* < 0.01]. Moreover, statistically significant differences were found between the health literacy scores based on ethnicity, with the Sindhi-speaking population having the highest scores at 56.0 (41.5–62), followed by Urdu-speaking at 41.0 (32.7–52.2) and Kashmiri, Punjabi and Pashtun and Hindko speaking at 36.0 (28–40), 35.0 (28.0–43), 33.5 (26.2–45) and 32.0 (28–40), *p* < 0.05. The impact of demographics and clinical characteristics on health literacy is given in Table 6.

**Table 6:** Comparison of demographic and clinical characteristics across health literacy, illness perception, and medication adherence total scores in hemodialysis patients

Variables	Health literacy		Illness perception		Medication adherence	
	Median (IQR)	*p*-value	Median (IQR)	*p*-value	Median (IQR)	*p*-value
Age (years)^b^		0.927		0.095		**0.001**
18–30	34.5 (28.0–43.0)		48.0 (46.0–53.0)		5.75 (3.75–7.50)	
31–45	36.0 (28.0–45.5)		51.0 (47.0–55.0)		5.50 (3.50–7.00)	
46–60	36.0 (29.0–42.7)		48.5 (44.0–53.0)		7.00 (4.75–7.75)	
≥ 61	36.0 (28.0–46.0)		49.0 (43.0–54.7)		7.00 (5.56–8.00)	
Gender^a^		0.469		**0.002**		0.790
Male	36.0 (28.0–45.0)		47.0 (43.2–53.0)		6.75 (4.50–7.75)	
Female	36.0 (28.0–44.0)		50.0 (46.0–54.0)		6.75 (4.75–7.75)	
Marital status ^b^		**0.049**		0.687		0.329
Single	37.0 (29.5–46.5)		49.0 (46.0–54.0)		6.00 (3.75–7.50)	
Married	36.0 (28.0–45.0)		49.0 (45.0–54.0)		6.75 (4.50–7.75)	
Others (divorced, widowed and widower)	32.0 (23.5–40.0)		49.0 (43.7–53.2)		6.75 (4.87–8.00)	
Qualification^b^		**0.000**		**0.020**		0.340
Uneducated	32.0 (24.0–40.0)		50.0 (45.7–55.0)		6.75 (4.50–7.75)	
Secondary education	33.0 (27.0–40.0)		49.0 (44.0–54.0)		6.75 (4.75–7.75)	
Tertiary education	44.5 (36.0–54.0)		47.5 (45.0–52.0)		6.00 (3.93–7.75)	
Current employment status^b^		0.321		**0.036**		0.314
Un-employed	35.0 (28.0–42.0)		49.0 (46.0–54.2)		6.00 (4.50–7.50)	
Employed	36.0 (32.0–45.0)		46.0 (43.0–51.0)		6.25 (3.75–7.00)	
Others (retried, self-employed and housewives)	36.0 (28.0–46.0)		49.0 (45.0–53.5)		6.75 (4.75–7.75)	
Family income^b^		**0.031**		**0.021**		**0.036**
10,000–30,000	36.0 (28.0–41.0)		52.0 (47.0–56.0)		5.50 (3.50–7.00)	
31,000–50,000	34.5 (28.0–42.0)		49.0 (45.0–55.0)		6.62 (4.75–7.75)	
51,000–100,000	37.0 (28.0–48.0)		48.0 (45.0–52.0)		6.75 (4.75–8.00)	
> 100,000	39.0 (33.0–55.7)		46.5 (41.5–50.7)		6.37 (2.87–7.75)	
Years on dialysis^b^		**0.000**		0.700		**0.016**
≤ 1	33.0 (26.0–40.5)		49.0 (45.0–54.0)		7.00 (4.87–8.00)	
2–3	37.5 (31.0–49.7)		49.0 (46.0–53.0)		6.25 (4.06–7.00)	
≥ 4	37.0 (29.5–49.5)		48.0 (45.0–54.0)		6.00 (3.62–7.75)	
Frequency of hemodialysis^b^		0.790		0.117		**0.007**
Once a week	36.5 (31.7–40.0)		48.0 (43.7–54.5)		6.25 (4.75–7.81)	
Twice a week	36.0 (28.0–45.0)		49.0 (45.0–53.0)		6.75 (4.75–7.75)	
Thrice a week	36.0 (30.0–48.0)		51.0 (47.0–57.0)		5.00 (3.00–7.00)	
Daily pills burden^b^		0.063		0.268		0.798
1–5	36.0 (28.0–44.0)		49.0 (45.0–53.0)		6.75 (4.75–7.75)	
6–10	37.0 (29.0–48.0)		50.0 (46.0–54.5)		6.75 (4.50–7.75)	
> 10	32.0 (24.0–42.2)		49.0 (44.0–53.2)		6.50 (3.50–7.56)	
Comorbidity^b^		0.067		**0.023**		0.801
None	38.5 (30.5–47.7)		47.0 (44.0–52.0)		5.75 (4.00–7.50)	
1 disease	36.0 (29.0–45.0)		48.0 (43.0–53.0)		6.75 (4.75–7.75)	
2–3 diseases	36.0 (28.0–45.0)		50.0 (46.0–54.0)		6.75 (4.50–7.75)	
4 diseases	30.50 (27–36)		49.50 (43.75– 54.25)		7.25 (4.50–8)	
Ethnicity^b^		**0.002**		**0.042**		0.430
Urdu speaking	41.0 (32.7–52.2)		47.0 (41.7–51.0)		6.00 (4.75–7.75)	
Punjabi speaking	35.0 (28.0–43.0)		50.0 (45.0–55.0)		6.75 (4.50–7.87)	
Pashtun speaking	33.5 (26.2–45.0)		49.0 (43.7–52.2)		5.62 (2.18–7.06)	
Sindhi speaking	56.0 (41.5–62.0)		48.0 (43.5–51.0)		5.75 (4.12–7.25)	
Hindko speaking	32.0 (28.0–40.0)		48.0 (42.5–53.0)		6.50 (4.62–7.00)	
Kashmiri speaking	36.0 (28.0–40.0)		51.0 (46.0–57.0)		6.75 (4.75–7.50)	

Bold values indicate results that are statistically significant (*p* 0.05)

^a^Statistical significance of differences calculated using the Mann–Whitney *U* test

^b^Statistical significance of differences calculated using the Kruskal–Wallis test

### Impact of demographic and clinical parameters on illness perception of the patients

Statistically significant differences in illness perception scores were observed between different demographic categories of participants. The mean illness perception scores were higher for females than males (50.3 ± 6.4 vs 48.2 ± 7.3, *p* < 0.005]. The mean illness perception scores of uneducated patients were significantly higher than those who had acquired secondary/high school education followed by higher education (college/bachelor/postgraduate) (50.6 ± 7.3 vs 49.6 ± 7.0 and 48.0 ± 6.3, *p* < 0.05). The mean illness perception scores of unemployed and retired/self-employed/housewives were significantly higher than employed patients (50.1 ± 6.6 and 49.4 ± 7.0 vs 46.9 ± 6.1, *p* < 0.05). The mean illness perception scores were higher for patients having 2–3 comorbid diseases as compared to those having no comorbidity, 1 and 4 comorbidities (50.3 ± 7.0 vs 47.2 ± 5.3, 48.1 ± 6.8 and 49.7 ± 7.3, *p* < 0.05). The illness perception scores varied significantly by ethnicity, with Kashmiri-speaking populations having higher mean scores than Punjabi followed by Pashtun speaking followed by Hindko, and Sindhi speaking with lowest scores for Urdu-speaking populations (Table 6).

### Impact of demographic and clinical parameters on medication adherence of the patients.

The medication adherence scores were significantly higher for patients aged 40–60 and > 61 years as compared to the patients aged 31–45 and 18–30 years with median values of (7.00 (4.75–7.75) and 7.00 (5.56–8.00) vs 5.75 (3.75–7.50), and 5.50 (3.50–7.00), *p* < 0.001). The medication adherence was significantly higher in patients with less than one year of dialysis as compared to those with 2–3 or more than 4 years with median values of [7.00 (4.87–8.00) vs. 6.25 (4.06–7.00) and 6.00 (3.62–7.75)]. However, patients having dialysis twice a week had higher medication adherence as compared to patients having dialysis thrice and once a week with mean values of [6.75 (4.75–7.75) vs. 6.25 (4.75–7.81) and 5.00 (3.00–7.00)]. The impact of demographics and clinical characteristics on medication adherence is given in Table 6.

### Association of demographic and clinical parameters with health literacy of the patients

Binary logistic regression was conducted to assess the association of health literacy with demographic variables. The patients aged between 18 and 30 years had significantly lower health literacy as compared to the patients of age ≥ 61 years [OR = 0.22 (95% CI 0.07–0.69, *p* = 0.009] which suggests that for every 1-year decrease in age, the likelihood of adequate health literacy also decreased. Similarly, HD patients with lower educational qualifications (Uneducated or Primary/Secondary/High school) were significantly more likely to have inadequate health literacy compared to those with College/Bachelors/Postgraduate education (Uneducated: OR 0.20, 95% CI 0.10–0.43, *p* < 0.001; Primary/Secondary/High school: OR 0.20, 95% CI 0.10–0.37, *p* < 0.001). No significant association was found between adequate health literacy and other demographic characteristics (Table 7).

**Table 7:** Multivariate binary logistic regression analysis to find factors associated with health literacy and illness perception among hemodialysis patients

Characteristics	Health literacy		Illness perception	
	OR (95% CI)	*p*-value	OR (95% CI)	*p*-value
Age (years)				
18–30	0.22 (0.07–0.69)	**0.009**	0.48 (0.16–1.43)	0.192
31–45	0.97 (0.44–2.11)	0.944	1.12 (0.52–2.41)	0.762
46–60	1.18 (0.64–2.15)	0.583	0.82 (0.46–1.47)	0.514
≥ 61	Reference	–	Reference	–
Gender				
Male	0.99 (0.58–1.70)	0.990	0.55 (0.33–0.92)	**0.024**
Female	Reference	–	Reference	–
Marital status				
Single	1.46 (0.39–5.42)	0.571	3.02 (0.83–10.9)	0.093
Married	0.92 (0.40–2.13)	0.860	1.54 (0.66–3.61)	0.317
Others (divorced, widowed and widower)	Reference	–	Reference	–
Qualification				
Uneducated	0.20 (0.10–0.43)	**0.000**	1.55 (0.79–3.04)	0.200
Secondary education	0.20 (0.10–0.37)	**0.000**	1.31 (0.75–2.29)	0.327
Tertiary education	Reference	–	Reference	–
Current employment status				
Un-employed	1.06 (0.53–2.14)	0.855	1.03 (0.52–2.03)	0.923
Employed	1.42 (0.57–3.53)	0.449	0.67 (0.27–1.66)	0.396
Others (retried, self-employed and housewives)	Reference	–	Reference	–
Family income (Pakistani Rupees)				
10,000–30,000	2.81 (0.55–14.3)	0.214	3.27 (0.65–16.5)	0.150
31,000–50,000	1.50 (0.32–6.88)	0.597	2.39 (0.52–10.8)	0.257
51,000–100,000	1.96 (0.43–8.89)	0.381	1.68 (0.37–7.54)	0.494
> 100,000	Reference	–	Reference	–
Years on dialysis				
≤ 1	0.64 (0.36–1.14)	0.131	1.30 (0.744–2.29)	0.353
2–3	1.14 (0.60–2.13)	0.681	1.21 (0.66–2.22)	0.519
≥ 4	Reference	–	Reference	–
Frequency of hemodialysis				
Once a week	1.50 (0.43–5.13)	0.518	0.38 (0.11–1.26)	0.116
Twice a week	0.87 (0.40–1.90)	0.735	0.57 (0.26–1.24)	0.158
Thrice a week	Reference	–	Reference	–
Daily pills burden				
1–5	1.08 (0.54–2.16)	0.810	1.70 (0.88–3.31)	0.113
6–10	1.44 (0.74–2.79)	0.277	1.76 (0.94–3.33)	0.077
> 10	Reference	–	Reference	–
Comorbidity				
None	9.43 (1.32–67.3)	**0.025**	0.23 (0.03–1.45)	0.119
One disease	4.17 (0.99–17.5)	0.051	0.52 (0.13–2.04)	0.357
2–3 diseases	3.04 (0.74–12.4)	0.121	0.74 (0.19–2.81)	0.668
Four diseases	Reference	–	Reference	–
Ethnicity				
Urdu speaking	2.20 (0.80–6.05)	0.124	0.41 (0.16–1.07)	0.071
Punjabi speaking	0.78 (0.34–1.80)	0.566	0.79 (0.35–1.78)	0.582
Pashtun speaking	0.74 (0.25–2.14)	0.580	0.61 (0.21–1.75)	0.359
Sindhi speaking	1.12 (0.09–13.6)	0.925	0.61 (0.08–4.69)	0.638
Hindko speaking	0.58 (0.19–1.80)	0.351	0.54 (0.18–1.63)	0.278
Kashmiri speaking	Reference	–	Reference	–
Medication adherence				
Low	0.68 (0.37–1.26)	0.227	1.57 (0.88–2.82)	0.124
Medium	1.04 (0.56–1.93)	0.887	0.97 (0.54–1.77)	0.943
High	Reference	–	Reference	–

Bold values indicate results that are statistically significant (*p* 0.05)

### Association of demographic and clinical parameters on illness perception of the patients

Male hemodialysis patients were significantly less likely to have a positive illness perception than female patients (OR 0.55, 95% CI 0.33–0.92, p = 0.024). No significant association was found between illness perception and other demographic and clinical variables (Table 7).

### Association of health literacy, illness perception, and demographic variables with medication adherence

Both the health literacy and illness perception scores did not have a statistically significant association with medication adherence (OR 1.01, 95% CI − 0.06 to 0.02, *p* = 0.210) and (OR 0.98, 95% CI − 0.04 to 0.01, *p* = 0.227), respectively. The patients aged 18–30 years had significantly lower odds of medication adherence compared to those aged ≥ 61 (OR 0.39, 95% CI − 1.87 to − 0.004, *p* = 0.049). Similarly, patients aged 31–45 had significantly lower odds of medication adherence than those of ≥ 61 (OR 0.50, 95% CI − 1.35 to − 0.01, *p* = 0.044). No significant differences were observed in medication adherence across various demographic and clinical characteristics among hemodialysis patients (Table 8).

**Table 8:** Multivariate ordinal logistic regression analysis of predictors of medication adherence among hemodialysis patients

Characteristics	OR (95% CI)	*p*-value
HL total score	1.01 (− 0.06 to 0.02)	0.210
IP total score	0.98 (− 0.04 to 0.01)	0.227
Age (years)		
18–30	0.39 (− 1.87 to − 0.04)	**0.049**
31–45	0.50 (− 1.35 to − 0.01)	**0.044**
46–60	0.80 (− 0.72 to 0.02)	0.403
≥ 61	Reference	–
Gender		
Male	1.18 (− 0.27 to 0.61)	0.462
Female	Reference	–
Marital status		
Single	1.59 (− 0.63 to 1.56)	0.405
Married	0.95 (− 0.75 to 0.66)	0.902
Others (divorced, widowed and widower)	Reference	–
Qualification		
Uneducated	1.38 (− 0.29 to 0.94)	0.307
Secondary education	1.57 (− 0.05 to 0.96)	0.078
Tertiary education	Reference	–
Current employment status		
Un-employed	0.87 (− 0.73 to 0.45)	0.650
Employed	0.77 (− 1.00 to 0.48)	0.495
Others (retried, self-employed and housewives)	Reference	–
Family income (Pakistani Rupees)		
10,000–30,000	0.47 (− 2.06 to 0.58)	0.272
31,000–50,000	0.90 (− 1.31 to 1.11)	0.869
51,000–10,0000	0.84 (− 1.36 to 1.02)	0.780
> 100,000	Reference	–
Years on dialysis		
≤ 1	1.46 (− 0.11 to 0.87)	0.128
2–3	0.92 (− 0.60 to 0.44)	0.769
≥ 4	Reference	–
Frequency of hemodialysis		
Once a week	1.38 (− 0.74 to 1.39)	0.554
Twice a week	2.05 (0.01 to 1.42)	**0.044**
Thrice a week	Reference	–
Daily pills burden		
1–5	1.11 (− 0.47 to 0.68)	0.721
6–10	1.09 (− 0.47 to 0.65)	0.757
> 10	Reference	–
Comorbidity		
None	0.73 (− 1.88 to 1.26)	0.701
One disease	0.86 (− 1.28 to 1.00)	0.811
2–3 diseases	0.82 (− 1.32 to 0.92)	0.731
Four diseases	Reference	–

Bold values indicate results that are statistically significant (*p* 0.05)

## Discussion

To the best of our knowledge, this is the first study to comprehensively assess the interplay between health literacy, illness perception, sociodemographic factors, and their combined impact on medication adherence in hemodialysis patients in Pakistan. More than half of the study patients had adequate health literacy and positive illness perception, but most patients had poor medication adherence. The study found a very weak negative correlation between health literacy and illness perception, a negative correlation between illness perception and medication adherence, and a positive correlation between health literacy and medication adherence, but none were statistically significant. Further, demographic factors such as education, marital status, number of years on dialysis, family income, and ethnicity had a significant association with health literacy. Similarly, gender, education, comorbidity status, family income, employment, and ethnicity had a significant impact on illness perception. Age, family income, years on dialysis, and frequency of dialysis had a significant influence on medication adherence.

There were no significant associations between health literacy, illness perception, and medication adherence. The lack of association between health literacy and illness perception among CKD patients observed in this study was consistent with the findings from a similar study on diabetes [44] but contrary to some other studies on hypertension and COPD [45, 46]. However, a weak negative correlation existed between health literacy and illness perception, which suggests that patients with higher health literacy were less likely to have a pessimistic view of their illness. Similarly, health literacy had no impact on medication adherence. However, it may have an indirect impact on patients’ self-care behavior. Studies on intermittent claudication and dialysis patients reported that individuals with higher health literacy exhibit lower emotional responses to their illness and higher self-efficacy in disease management and adherence to recommendations [24, 47]. Additionally, most of the patients in our study reported that their medications were managed by their family members or caregivers, which could have enhanced adherence measures, leading to a lack of correlation between health literacy and adherence.

The patients also reported that either they had a chronic history of diabetes and hypertension or a family history of CKD. These factors might have increased their health literacy and medication adherence rates as the results showed that half of the study population found it easy to read and understand patient education material. Furthermore, our study findings indicated that 45.1% of patients face challenges understanding written information, appointment forms, and medication slips and lack confidence in filling in their forms. These findings align with studies conducted In the USA and Australia [48–50]. For instance, in research assessing health literacy among patients with CKD, over 40% exhibited inadequate health-related attitudes [49]. Similarly, in the study, which included hemodialysis patients, about 32.3% of participants exhibited inadequate health literacy [50]. These findings were consistent with research showing that patients with higher health literacy have higher self-care behavior and higher self-efficacy in adherence to medication regimen and lifestyle management, which leads to a better quality of life and disease prognosis [51–53]. Therefore, targeted educational interventions (cognitive-behavioral interventions and structured educational programs) and healthcare professional-led interventions (Nephrologists, pharmacists, nurses, and dieticians) are needed among hemodialysis patients in Pakistan to improve their health literacy and illness perceptions.

The sociodemographic and clinical characteristics of the study population significantly impacted their health literacy, illness perceptions, and medication adherence. The results indicated that lower education levels were associated with lower health literacy and negative illness perception, which suggests that patients from lower educational backgrounds require additional support. Moreover, health literacy was lower in patients with no comorbid disease than in patients with comorbid diseases. Additionally, the patients undergoing more frequent dialysis had higher health literacy. This finding is consistent with Rakhshan et al.’s results, where frequent interactions with healthcare providers were linked to better adherence [54]. Another study reported that home hemodialysis patients had better health literacy than in-center hemodialysis patients, as they received personalized communication from healthcare providers [55]. There were Significant differences in health literacy and illness perceptions between different ethnic groups, consistent with the findings in the dialysis population by Ibelo et al. [56] and in the general population by Yiğitalp et al. [57].

In addition to their impact on health literacy and illness perception, certain demographic factors may act as barriers or facilitators to medication adherence. Young age was a barrier to medication adherence as the younger patients (18–30) had lower health literacy and were less likely to adhere to medication regimens as compared to older patients (> 61 years). This finding aligns with previous research that reported lower adherence in younger individuals due to their inexperience with healthcare systems, high workload or education commitments, and peer-related stigma [58, 59]. This finding implies that younger patients may require additional support and tailored interventions to adhere to their medication. The following interventions should be suggested to improve their adherence, including parental and family education,

SMS and WhatsApp reminders, peer support groups, storytelling and patient role models, motivational interviewing, and behavioral reinforcement. Similarly, the frequency of healthcare visits facilitated medication adherence as patients receiving hemodialysis more frequently were likelier to have higher medication adherence. The higher frequency of hemodialysis was associated with higher health literacy levels, indicating that health literacy likely indirectly affects medication adherence.

On the other hand, monthly family income substantially correlated with medication adherence; as family income increased, the patients demonstrated a greater likelihood of adhering to their prescribed prescriptions. This result aligns with a prior study indicating that socioeconomic level significantly correlates with medication adherence [60, 61]. A qualitative study conducted in Australia to investigate factors influencing medication adherence in ESRD patients revealed that financial restrictions related to medication non-adherence [62]. Income status has been associated with non-adherence in multiple studies involving renal patients. Moreover, low socioeconomic position has been markedly correlated with medication non-adherence in patients with chronic kidney disease (CKD) [63]. In underdeveloped nations, most CKD patients lack adequate health insurance, resulting in costly medical care that adversely impacts their adherence to treatment protocols. A significant number of CKD patients in underdeveloped nations cease therapy after commencing dialysis due to budgetary limitations. [64, 65]. Qualitative research conducted by Lindberg & Lindberg [66] highlighted forgetfulness and intricate schedules resulting from polypharmacy as the primary barriers to non-adherence.

### Future implication

This study highlights the need for tailored educational programs and structured interventions to improve medication adherence, particularly among patients with lower education levels, younger age groups, and diverse ethnic backgrounds. In addition to frequent and personalized communication between patients and healthcare providers, we recommend following targeted interventions such as structured educational programs (interactive workshops, visual aids), digital health solutions (mHealth applications, SMS reminders, and telehealth counseling), cognitive-behavioral interventions (motivational interviewing, problem-solving therapy, and behavioral reinforcement strategies) and healthcare providers training (patient center communication skill). Such interventions not only enhance patients’ education level about disease and treatment but also reshape patients’ perception of illness and adherence behavior. Future research may focus on longitudinal studies to understand the causative relationships between factors affecting medication adherence. Additionally, the role of ethnicity in shaping illness perception and health literacy needs further exploration using larger, more diverse cohorts. Evaluating the effectiveness of targeted educational interventions on adherence across different genders, ages, and ethnicities can inform the development of inclusive health literacy policies and support strategies, particularly for vulnerable groups.

### Strengths and limitations

This is the first study to elaborate on the interplay between health literacy, illness perceptions, and medication adherence among Pakistani CKD patients. However, a few limitations remain evident in our investigation. First, the study’s cross-sectional design did not allow for interpreting causal relationships between variables, emphasizing the need for longitudinal studies in future research. Second, the reliance on the subjective assessment of medication adherence may have introduced social desirability bias, potentially skewing responses. Objective measures such as pharmacy refill records could mitigate this issue in future studies. Finally, a direct comparison with equivalent studies is not possible, as no similar studies on HD patients from Pakistan are available, highlighting a gap in the literature that future research should address.

## Conclusion

This study found that nearly half of the hemodialysis patients in Pakistan patients demonstrate low medication adherence, inadequate health literacy, and negative perceptions of their illness, highlighting the urgent need for targeted interventions. This may be attributed to a low literacy rate and healthcare professionals’ lack of patient-centered education and counseling. To address these issues, healthcare professionals in hemodialysis units should focus on personalized interventions to bridge the health literacy gap and improve medication adherence levels, particularly in low-resource healthcare settings. Since adherence is a multifactorial problem, a collective approach involving patients, families, and healthcare professionals (doctors, pharmacists, and nurses) is needed to enhance the population's health literacy and adherence to medications. Additionally, policymakers should take these aspects into account when formulating comprehensive educational and interventional programs to enhance patient adherence to medicine. Targeted cognitive-behavioral interventions and structured educational programs such as patient counseling, mHealth solutions, and peer support may yield better outcomes in enhancing adherence.

## Supplementary Information

Below is the link to the electronic supplementary material.

Supplementary file1 (DOCX 23 KB)

## Data Availability

Data is provided within the manuscript or supplementary information files.

## Abbreviations

BIPQ: Brief illness perception questionnaire
CKD: Chronic kidney disease
ESRD: End-stage renal disease
GFR: Glomerular filtration rate
HD: Hemodialysis
HL: Health literacy
HLQ: Health Literacy Questionnaire
IP: Illness perception
MA: Medication adherence
MMARS- 8: 8-Item Morisky medication adherence rating scale
RRT: Renal replacement therapy
STROBE: Strengthening the Reporting of Observational Studies in Epidemiology
WHO: World Health Organization
