# Supplementary material for: Health literacy, illness perception, and their association with medication adherence in end-stage renal disease
Source: Int Urol Nephrol. 2025 Apr 4;57(9):2979–94. doi: 10.1007/s11255-025-04472-8 (PMC12350584; doi:10.1007/s11255-025-04472-8)
Supplement: Supplementary file 1 — Supplementary file1 (DOCX 23 KB) [file 11255_2025_4472_MOESM1_ESM.docx]

**INVESTIGATE THE IMPACT OF HEALTH LITERACY AND ILLNESS PERCEPTION ON MEDICATION ADHERENCE IN HEMODIALYSIS PATIENTS: A CROSS-SECTIONAL STUDY**

**INFORMED CONSENT FORM:**

You are invited to participate in a research study that aims to investigate the impact of illness perception and health literacy on medication adherence among hemodialysis patients. Your voluntary participation in this study will involve answering questions related to your illness perception, health literacy, and medication adherence. Your personal information will be kept confidential. Participation is entirely voluntary, and you may withdraw at any time without consequences. Your input will contribute to valuable insights for improving healthcare for hemodialysis patients. I agree to participate in this research study.

**Participant's Signature:** _________________  **Date:** ______________

**SECTION:1 DEMOGRAPHIC INFORMATION**

1. **Age:** ☐ 18–30 years ☐ 31–45 years ☐ 46–60 years ☐ ≥ 61 years
2. **Gender:** ☐ Male ☐ Female
3. **Marital status:** ☐ Single ☐ Married ☐ Others (Divorced, Widowed, Widower)
4. **Educational Background:** ☐ Uneducated ☐ Primary School/Secondary School/ College

☐ Bachelor's Degree/ Post Graduate

1. **Current employment status:** ☐ Un-employed ☐ Employed ☐ Others (Retired/self-employed/House Wife)
2. **Income Per Month (PKR)*:** ☐ 10,000-30,000 ☐ 31,000–50,000

☐ 51,000 –100,000 ☐ > 100,000

1. **Years on dialysis:** ☐ ≤ 1 year ☐ 2–3 years ☐ ≥ 4 years
2. **Frequency of Hemodialysis:** ☐ One time a week ☐ Two times a week ☐ Three times a week
3. **Daily pills burden:** ☐ 1-5 ☐ 6-10 ☐ > 10
4. **Comorbidity:** ☐ None ☐ 1-disease ☐ 2-3 disease ☐ 4 disease
5. **Ethnicity:** ☐ Urdu speaking ☐ Punjabi ☐ Pashtun ☐ Sindhi ☐ Hindko ☐ Kashmiri

**SECTION:2 HEALTH LITERACY SCREENING QUESTIONNAIRE**

**For the following questions, please choose one option that best corresponds to your views.**

1. **How often are appointment slips written in a way that is easy to read and understand?**

☐ Always ☐ Often ☐ Sometimes ☐ Occasionally ☐ Never

1. **How often are medical forms written in a way that is easy to read and understand?**

☐ Always ☐ Often ☐ Sometimes ☐ Occasionally ☐ Never

1. **How often are medication labels written in a way that is easy to read and understand?**

☐ Always ☐ Often ☐ Sometimes ☐ Occasionally ☐ Never

1. **How often are patient educational materials written in a way that is easy to read and understand?**

☐ Always ☐ Often ☐ Sometimes ☐ Occasionally ☐ Never

1. **How often are hospital or clinic signs difficult to understand?**

☐ Always ☐ Often ☐ Sometimes ☐ Occasionally ☐ Never

1. **How often are appointment slips difficult to understand?**

☐ Always ☐ Often ☐ Sometimes ☐ Occasionally ☐ Never

1. **How often are medical forms difficult to understand and fill out?**

☐ Always ☐ Often ☐ Sometimes ☐ Occasionally ☐ Never

1. **How often are directions on medication bottles difficult to understand?**

☐ Always ☐ Often ☐ Sometimes ☐ Occasionally ☐ Never

1. **How often do you have difficulty understanding the written information your health care provider (like a doctor, nurse, or nurse practitioner) gives you?**

☐ Always ☐ Often ☐ Sometimes ☐ Occasionally ☐ Never

1. **How often do you have problems getting to your clinic appointments at the right time because of difficulty understanding written instructions?**

☐ Always ☐ Often ☐ Sometimes ☐ Occasionally ☐ Never

1. **How often do you have problems completing medical forms because of difficulty understanding the instructions?**

☐ Always ☐ Often ☐ Sometimes ☐ Occasionally ☐ Never

1. **How often do you have problems learning about your medical condition because of difficulty understanding written information?**

☐ Always ☐ Often ☐ Sometimes ☐ Occasionally ☐ Never

1. **How often are you unsure of how to take your medication(s) correctly because of problems understanding written instructions on the bottle label?**

☐ Always ☐ Often ☐ Sometimes ☐ Occasionally ☐ Never

1. **How confident are you in filling out medical forms by yourself?**

☐ Always ☐ Often ☐ Sometimes ☐ Occasionally ☐ Never

1. **How confident do you feel you can follow the instructions on the label of a medication bottle?**

☐ Always ☐ Often ☐ Sometimes ☐ Occasionally ☐ Never

1. **How often do you have someone (like a family member, friend, hospital/clinic worker, or caregiver) help you read hospital materials?**

☐ Always ☐ Often ☐ Sometimes ☐ Occasionally ☐ Never

**SECTION:3 BRIEF ILLNESS PERCEPTION SCALE**

**BRIEF ILLNESS PERCEPTION SCALE**

**For the following questions, please choose the number that best corresponds to your views.**

1. **How much does your illness affect your life?**

0 1 2 3 4 5 6 7 8 9 10

No affect Severely affects

at all my life

1. **How long do you think your illness will continue?**

0 1 2 3 4 5 6 7 8 9 10

A very

short time Forever

1. **How much control do you feel you have over your illness?**

0 1 2 3 4 5 6 7 8 9 10

Absolutely Extreme amount

no control of control

1. **How much do you think your treatment can help your illness?**

0 1 2 3 4 5 6 7 8 9 10

Not at all Extremely helpful

1. **How much do you experience symptoms from your illness?**

0 1 2 3 4 5 6 7 8 9 10

No symptoms

at all Many severe symptoms

1. **How concerned are you about your illness?**

0 1 2 3 4 5 6 7 8 9 10

Not at

all concerned Extremely concerned

1. **How well do you feel you understand your illness?**

0 1 2 3 4 5 6 7 8 9 10

Don’t understand Understand

at all very clearly

1. **How much does your illness affect you emotionally? (e.g., does it make you angry, scared, upset or depressed?)**

0 1 2 3 4 5 6 7 8 9 10

Not at all Extremely

affected emotionally affected emotionally

1. **Please list in rank order the three most important factors that you believe caused your illness.**

**The most important causes for me: -**

1. __________________________________

2. __________________________________

3. __________________________________

**SECTION:4 MORISKY MEDICATION ADHERENCE SCALE**

**Medication Adherence Screening Questions**

| **S.NO.** | **QUESTIONS** | **YES** | **NO** |
| --- | --- | --- | --- |
| **1.** | **Do you sometimes forget to take your medication?** |  |  |
| **2.** | **Over the past 2 weeks, were there any days that you did not take your medication?** |  |  |
| **3.** | **Have you ever cut back or stopped taking your medication without telling your doctor because you felt worse when you took it?** |  |  |
| **4.** | **When you travel or leave home, do you sometimes forget to bring your medication?** |  |  |
| **5.** | **Did you take your medication yesterday?** |  |  |
| **6.** | **When you feel that your health concern is under control, do you sometimes stop taking your medication?** |  |  |
| **7.** | **Taking medication every day is a real inconvenience for some people. Do you ever feel hassled about sticking to your treatment plan?** |  |  |
| **8.** | **How often do you have difficulty remembering to take all your medication?** | | |
| **NEVER/RARELY,** | |  | |
| **ONCE IN A WHILE,** | |  | |
| **SOMETIMES,** | |  | |
| **USUALLY,** | |  | |
| **ALL THE TIME,** | |  | |

**THANK YOU FOR YOUR TIME AND COOPERATION IN FILLING OUT THIS QUESTIONNAIRE. YOUR VALUABLE INPUT IS GREATLY APPRECIATED AND WILL CONTRIBUTE TO OUR RESEARCH EFFORTS. 🙂🙂**
